# Executive functions and brain morphology of male and female dominant and subordinate cichlid fish

**DOI:** 10.1002/brb3.3484

**Published:** 2024-04-29

**Authors:** Angelo Guadagno, Zegni Triki

**Affiliations:** ^1^ Behavioural Ecology Division, Institute of Ecology and Evolution University of Bern Bern Switzerland

**Keywords:** associative learning, cognitive flexibility, inhibitory control, object permanence, size‐based hierarchy, social pressures, working memory

## Abstract

**Background:**

Living in a social dominance hierarchy presents different benefits and challenges for dominant and subordinate males and females, which might in turn affect their cognitive needs. Despite the extensive research on social dominance in group‐living species, there is still a knowledge gap regarding how social status impacts brain morphology and cognitive abilities.

**Methods:**

Here, we tested male and female dominants and subordinates of *Neolamprologus pulcher*, a social cichlid fish species with size‐based hierarchy. We ran three executive cognitive function tests for cognitive flexibility (reversal learning test), self‐control (detour test), and working memory (object permanence test), followed by brain and brain region size measurements.

**Results:**

Performance was not influenced by social status or sex. However, dominants exhibited a brain–body slope that was relatively steeper than that of subordinates. Furthermore, individual performance in reversal learning and detour tests correlated with brain morphology, with some trade‐offs among major brain regions like telencephalon, cerebellum, and optic tectum.

**Conclusion:**

As individuals’ brain growth strategies varied depending on social status without affecting executive functions, the different associated challenges might yield a potential effect on social cognition instead. Overall, the findings highlight the importance of studying the individual and not just species to understand better how the individual's ecology might shape its brain and cognition.

## Graphical abstract

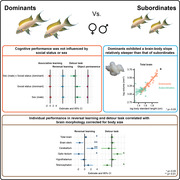

## 1 INTRODUCTION

Living in social groups poses various social challenges for individuals, such as sharing and defending territories and resources, mate choice, and bookkeeping (remembering partners’ behavior during past social interactions) (De Dreu & Triki, 2022; Lukas & Clutton‐Brock, 2018). One of the important challenges that group‐living animals face is adjusting behavior to their own position in a social dominance hierarchy (Leimar & Bshary, 2022; Strauss et al., 2022). In most social species, dominant individuals are older and physically more capable of defending their status and, hence, enjoy more privileges than low‐ranking younger subordinates. These privileges might include access to food, reproductive opportunities, and sometimes assistance from subordinates in caring for the offspring and defending the group against predators and intruders in cooperative breeding species (Bergmüller et al., 2005; Cant, 2012; Fernald, 2014). However, little is known about whether acquired privileges for the dominants would allow them to have fewer constraints on investing energy in neural tissue than subordinates and, hence, have improved cognitive abilities. Alternatively, changing social status might induce adaptive plasticity to face the new challenges without morphological modifications in the brain (Fernald, 2014).

Despite the interest in social dominance hierarchy (Leimar & Bshary, 2022; Strauss et al., 2022), to the best of our knowledge, only a few studies have looked either into the brain structure or cognitive abilities of dominant and subordinate individuals but failed to examine the important relationship between brain structure and cognition simultaneously in order to understand individual differences. For instance, the mushroom body calyx, a crucial structure in insect brains that plays a significant role in memory and learning, is more developed in dominant paper wasps than in subordinates (O'Donnell et al., 2007). In vertebrates, coupling MRI and fMRI scans helped to pinpoint throughout the whole brain the morphological neural correlates of social dominance and subordination in macaques, ruling out that all neural circuits are equally involved in social dominance hierarchy (Noonan et al., 2014). For the cognitive studies, dominant food caching chickadees display greater spatial learning and more efficient food caching than subordinates (Pravosudov et al., 2003). Similarly, dominant meadow voles and pheasants perform better in spatial learning tasks compared to the subordinates (Langley et al., 2018; Spritzer et al., 2004). In the African cichlid fish, *Astatotilapia burtoni*, Wallace et al. (2022) found that social status can result in different social competence abilities. Fish that ascended to a dominant status demonstrated improved use of social information, allowing them to perform optimal behaviors. Despite these valuable insights, there is still a gap in understanding how social dominance hierarchy affects brain morphology and cognitive performance. To address this, we need to assess not only one cognitive ability but multiple key abilities of dominant and subordinate individuals and examine the neural correlates of their performance.

Some key cognitive processes known as the main executive functions can play a pivotal role in survival and reproduction and have important fitness consequences in vertebrates (Barkley, 2012; Burkart et al., 2016). These complex executive function processes (cognitive flexibility, self‐control, and working memory) control several other cognitive subprocesses (Diamond, 2013; Miyake et al., 2000). For instance, cognitive flexibility helps to adjust behavior when demands shift, which makes it easier to adapt and thrive in changing environments (Uddin, 2021). Self‐control, which is often measured as inhibitory control (Beran, 2015; MacLean et al., 2014), requires resisting impulses in order to perform more goal‐oriented behaviors (Kabadayi et al., 2018; Köhler, 1925). Finally, working memory allows to hold temporarily information that is no longer perceptually present, guiding thus optimal decision‐making and behavior (Dudchenko et al., 2013; Read et al., 2022).

Fishes provide a suitable clade to study whether differences in social status correlate with differences in executive functions. Here, we used the African cichlid, *Neolamprologus pulcher*, as a study model. *N. pulcher* is a fish species with a complex social structure, making it ideal for investigating the impact of social status on brain morphology and executive function abilities. This species is a cooperative breeder that follows a size‐based social hierarchy (Hamilton et al., 2005; Heg et al., 2004). In this system, subordinates are smaller‐bodied fish that forgo their own reproduction and help larger‐bodied dominant breeders raise their offspring. In the wild, these fish form stable social groups composed of one larger dominant breeding pair and up to 20 smaller and younger subordinate helpers (Bergmüller et al., 2005; Taborsky, 2017; Wong & Balshine, 2011). In our study, we tested dominants and subordinates of captive‐bred *N. pulcher* in three executive function tests. After the tests, we collected their brains to assess the volume of the five main fish brain regions: telencephalon, optic tectum, hypothalamus, cerebellum and brain stem. We used the reversal learning paradigm as the standard test for cognitive flexibility in animal cognition studies (Ashton et al., 2018; Buechel et al., 2018; Deaner et al., 2006; Izquierdo et al., 2017; Triki & Bshary, 2021). For inhibitory control abilities, we used the cylinder task (MacLean et al., 2014; Triki et al., 2023). Finally, the third executive function test was an object permanence task. The task assesses if fish can memorize an object's location as it moves behind a screen and infer its continued existence when hidden (Triki et al., 2023).

Studies on various vertebrate species have shown that there are sex differences in specific cognitive performances and brain morphology, which can be attributed to selection based on different ecological needs (Choleris & Kavaliers, 1999; Cummings, 2018; Morand‐Ferron et al., 2016). The differences in brain development between males and females due to distinctive hormonal and neurohormonal pathways (Gemmell et al., 2019) might be the underlying mechanisms for both adaptive and nonadaptive sex differences (McEwen & Milner, 2017). In our current study, we tested both male and female cichlids. Our objective was to understand the social dominance hierarchy and its relationship with brain morphology and cognitive performance in these fish. We hypothesized that social dominance privileges would lead to increased investment in brain development and, hence, improved performance in executive functions. As for sex differences, our study is exploratory as the evidence is controversial in fish and can vary depending on the species being studied. According to an extensive review by Lucon‐Xiccato (2022) on sex differences in executive functions across species and taxa, the evidence is inconclusive, and it varies significantly from one species to another.

## 2 MATERIALS AND METHODS

### 2.1 Study animals and experimental set‐up

We ran our study between May and July 2023 at the Ethological Station of the University of Bern, Switzerland. We used sexually mature captive‐bred African cichlid, *Neolamprologus pulcher*, descendants of wild‐caught populations from Lake Tanganyika. From nine stock tanks of 400 L, we transferred 24 males and 24 females to individual experimental aquaria of 50 L equipped with a water filter, shelter and 3 cm layer of sand as enrichment. Half of the collected males and females were the largest individuals (dominants), while the other half were the smallest (subordinates) (Lerena et al., 2021) with a standard length larger than 3.5 cm. Sexual maturity in *N. pulcher* occurs at the age of about 1 year, with a standard length between 3 and 3.5 cm (Taborsky, 1985) (see also dissection note below). Water temperature was maintained at 27 ± 1°C, and the light:dark cycle was set at 13:11 h. The real identity of the tested fish was concealed with running numbers (#1, #2, etc.) to blind the experimenter and avoid potential subconscious observer bias in data collection.

In the stock tanks, fish received fish flakes 5 days a week and frozen zooplankton (that includes krill) once a week ad libitum. Once in the experimental tank, we fed fish on the first 3 days of acclimation with fish flakes. For the next 3 days, we habituated them to feed off 1 mL plastic pipettes delivering defrosted krill, which later served as food reward during the cognitive tests. Two fish did not eat from the pipette, so we returned them to their home stock tanks. Additionally, throughout the experiment, three fish were found dead, and some other fish did not systematically participate in all tests (see below), with one fish (#23) stopping completely to participate further after the associative learning. This led to sample size fluctuating from the original design across the tests (see Table S1 for detailed sample size per dataset).

### 2.2 Cognitive tests

The experimental set‐up and the cognitive test paradigms followed the protocols described by Triki et al. (2023). The experimental aquaria had housing and test compartments. To prevent isolation anxiety, the aquaria were placed next to each other so that the fish could see their neighbors in the housing compartment and not in the test compartment to avoid social learning during the tests. The experimenter used a see‐through and an opaque Plexiglas barrier (length × width, 24 × 22 cm) to isolate the fish in their housing compartment before each test trial. Lifting the opaque barrier, followed by the transparent barrier, allowed the fish to see what was in the test compartment before accessing the test paradigm. This procedure was used throughout the different cognitive tests.

#### 2.2.1 Color discrimination test (associative learning test)

We used as cues yellow and red plastic chips of 1.5 cm diameter. Half of the fish had yellow as the initial rewarding cue, while the other half had red. During the first 3 days, we presented the fish once a day with their corresponding rewarding colored chip with a defrosted krill placed on the top. In the following 3 days, we presented the cue and fish received the food reward only if they swam very close to the chip (within half a body length). We then glued the yellow and red chips on a see‐through Plexiglas support (length × width, 22 × 2.5 cm) allowing a fixed distance of 20 cm between the two cues. We offered the fish one acclimation trial with both cues presenting a food reward on the correct color. Three trials were conducted where fish only received a food reward if they approached the rewarding cue, facing it within half a body length.

During the test (Video S1), the experimenter rewarded the fish no matter whether they chose the correct color first or second. However, a “success” was only scored if the fish chose the correct color on the first attempt. Otherwise, if the fish explored the other color before approaching the correct one, we scored its performance as a “failure.” Some fish were relatively slow to perform, so we settled on a maximum of 15 min per trial. Fish received one test session per day (six trials), with the rewarding color being presented on the left or right side 50% of the time in a random sequence, with no more than three successive presentations on the same side. We considered a fish has successfully learned the cue‐reward association if they scored either six correct choices out of six consecutive trials in one session (6 out of 6 trials) or five correct choices out of six trials in two consecutive sessions (5/6 and 5/6 in two consecutive sessions). These learning criteria fit a learning probability significantly higher than the 50 % chance level of scoring correct (*p *< .05, with a binomial test). Once a fish reached the learning criterion, we started testing it in the reversal learning task in the following day. We ended the associative test when all fish successfully learned the cue‐reward association (11 sessions = 66 trials), except for one fish that even after 15 sessions (90 trials) did not learn (#23).

### 2.3 Reversal learning test

We reversed the cue‐reward contingency for those who learned the initial association by making the previously unrewarding color the new rewarding cue. We delivered a food reward only if the fish scored correctly, providing thus positive reinforcement in case of success and negative reinforcement (no food) in failure. Individual performance was evaluated using the same learning criteria as in the associative learning test. The objective was to end the test when at least 70% of the population learned the test successfully (Triki et al., 2023). However, after 144 trials, we had about 66% success, and we decided to end the test as the fish who did not reach the learning criterion were not improving further.

### 2.4 Detour test

In the detour test, we used a transparent Plexiglas cylinder (Triki et al., 2023, 2024) open on both sides (10 cm length and 8 cm diameter). We run 2 days of habituation, where we first habituated fish to feed off a green plastic chip (1.5 cm diameter), offering a defrosted krill in eight trials over 2 days. Afterward, we exposed the fish to the transparent cylinder for 4 h but with no food reward nor the green disc. During the test per se, we presented the fish with the cylinder with a food reward placed inside it. The food was placed on top of a green spot to eventually increase the salience of the food reward (Triki et al., 2023, 2024). To reach the reward, the fish had to detour the cylinder and swim inside. We scored performance as “success” if the fish detoured the cylinder without touching it (Video S2). Otherwise, if the fish bumps to the cylinder walls before retrieving the food, we scored the performance as “failure.” We allowed fish a maximum of 5 min to perform. We tested the fish over 3 days for a total of 16 trials: six trials on days 1 and 2 and four trials on day 3. Out of 43 tested fish, 33 participated in the detour test. The other 10 fish did not leave the home compartment in any of the 16 trials.

### 2.5 Object permanence test

As the object in this test, we used colored (yellow or red) plastic chips (1.5 cm diameter) glued on a see‐through Plexiglas handle (length × width, 22 × 2.5 cm). Fish had one trial acclimation with the object before the test, where they were given a food reward upon approaching or touching the object. For the test, we used an apparatus consisting of an opaque screen (22 × 6 cm) with see‐through Plexiglas glued to its back (22 × 6 cm), forming a T shape that created left and right spaces where to hide the object. The opaque screen prevented the fish from seeing the object once completely hidden by the experimenter, while the see‐through screen prevented access to the object if the fish followed the wrong path (see Triki et al., 2023). A test trial consisted of removing first the opaque divider, allowing fish to see but not access yet the test compartment. The experimenter then introduced the object in the middle of the test compartment and ensured the fish was facing the object before displacing and hiding it either on the left or right side. Within the first 10 seconds of having the object out of sight, we allowed the fish to enter the test compartment, and the experimenter recorded whether they followed the object's path successfully (Video S3). Upon locating the object successfully on the first attempt, the experimenter rewarded the fish with a krill. We controlled for potential side biases by displacing the object 50% of the time on the left and 50% on the right in random sequences with no more than three successive displacements on the same side. Over 3 days, fish received 16 test trials: four trials on day 1 and six trials on days 2 and 3. Of the 43 fish tested, 31 participated in the object permanence test. The other 12 fish did not leave the home compartment in any of the 16 trials.

### 2.6 Dissection and brain morphology measurements

We euthanized the fish with an overdose of tricaine methanesulfonate, MS‐222. We then measured their standard length (SL) and weight before fixing the whole bodies in 4% paraformaldehyde (PFA) at 4°C for 7 days. We collected and placed the brains in 2 % PFA at 4°C for another 3 days. We used Nikon SMZ1000 Microscope to take pictures of the dorsal, ventral, right lateral and left lateral panels of the brain. With the open‐access Fiji software (Schindelin et al., 2012), we estimated the length (L), width (W), and height (H) of the telencephalon, optic tectum, hypothalamus, cerebellum, and brain stem. We calculated the volume of each brain region by fitting their corresponding measurements in an ellipsoid function using the formula: Volume = (L × W × H) π / 6 (Triki, Granell‐Ruiz et al., 2022). The ellipsoid method is a low‐cost technique that allows for volumetric assessment of the main brain regions in fish for quantitative analysis (White & Brown, 2015).

During dissection, we examined the internal reproductive organs to verify that all tested fish had well‐developed ovaries and testes. It also served to confirm the accuracy of our visual inspection of the external genitalia and to avoid potential experimenter errors. Four fish were found to have such errors. After correcting the sex, we had 19 females (11 dominants, 8 subordinates) and 24 males (10 dominants and 14 subordinates).

### 2.7 Data analysis

We ran all the statistical analyses and generated the figures using the open‐access software R, version 4.2.1 (R Core Team, 2022).

#### 2.7.1 Cognitive performance data, social status, and sex

To test learning performance in the associative and reversal learning tests, we run two survival analyses with the Cox proportional hazards mixed models (*coxme*). We fitted as a response variable the success and failure combined with time as the number of sessions to reach learning. The models had social status (dominant vs. subordinate) and sex (male vs. female) as categorical predictors, while the stock tank identity was the random factor. For the detour and object permanence performances, we run two Bayesian Generalized Linear Mixed Models (BGLMM) with binomial error distribution. We fitted performance with a *cbind* function for number of successes and number of failures across the 16 test trials as a response variable. Social status and sex were the categorical variables, while stock tank identity was the random variable.

#### 2.7.2 Brain morphology data, social status, and sex

We fitted a set of Bayesian Linear Mixed Models (BLMM) to test for brain morphology. The response variable was one of the six brain measurements (in mm^3^) log‐transformed, that is, total brain, telencephalon, hypothalamus, optic tectum, cerebellum, and brain stem. The predictors were social status, sex and body size (log‐transformed and standardized SL in cm with the *scale* function) (Nakagawa et al., 2017), while stock tank identity was the random variable. We checked mathematically that our models did not have issues of collinearity between social status and body size affecting model estimates. To verify this, we used the Variance Inflation Factor (VIF) analyses and found that all values were lower than 3, indicating relatively low and negligible collinearity problems (Salmerón‐Gómez et al., 2020).

#### 2.7.3 Individual cognitive performance and brain morphology data

To test whether the brain measurements predicted individual cognitive performance, we ran a set of statistical mixed effects models where cognitive performance was the response variable, while brain measurements were the continuous predictors. Similar to the logic above, we run survival analyses (coxme) on learning data and BGLMMs on detour and object permanence data. The first set of models had log‐transformed and standardized total brain and body sizes as continuous predictors. The other set of tests had the five brain region sizes also log‐transformed and standardized as well as body size as continuous predictors. All models accounted for stock tank identity as a random variable

For further details, we provide a step‐by‐step R code and the corresponding data used to generate the findings (see the Data and Code accessibility statement).

## 3 RESULTS

**FIGURE 1:**
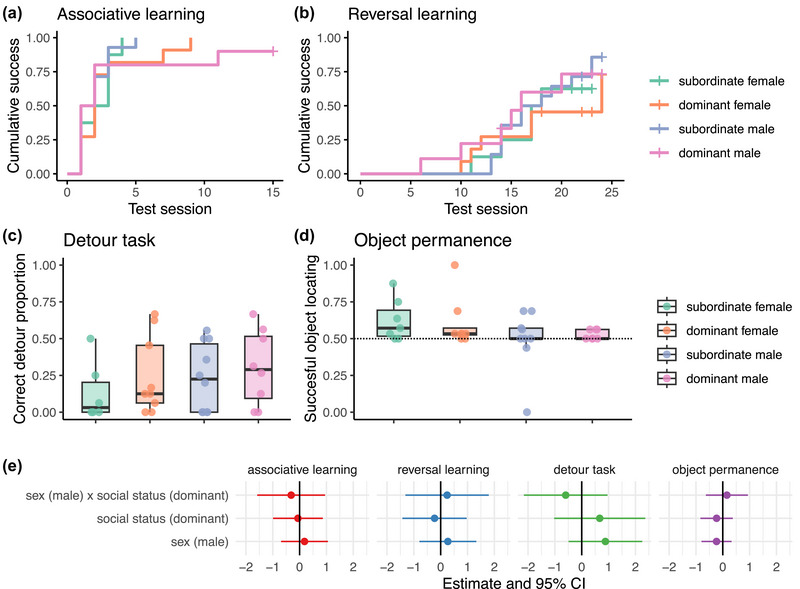
Performance in the cognitive tests. Cumulative success in (a) associative (*N* = 43) and (b) reversal learning (*N* = 42) tests. Boxplots of median, interquartile, and ranges of proportion of (c) correct detours (*N* = 33) and (d) successful object locating in object permanence (*N* = 31) tests. Dashed line in (d) indicates the 50% chance level of performing correctly. (e) Estimate and 95% confidence interval extracted from the statistical models for each cognitive performance as a function of social status and sex.

**FIGURE 2:**
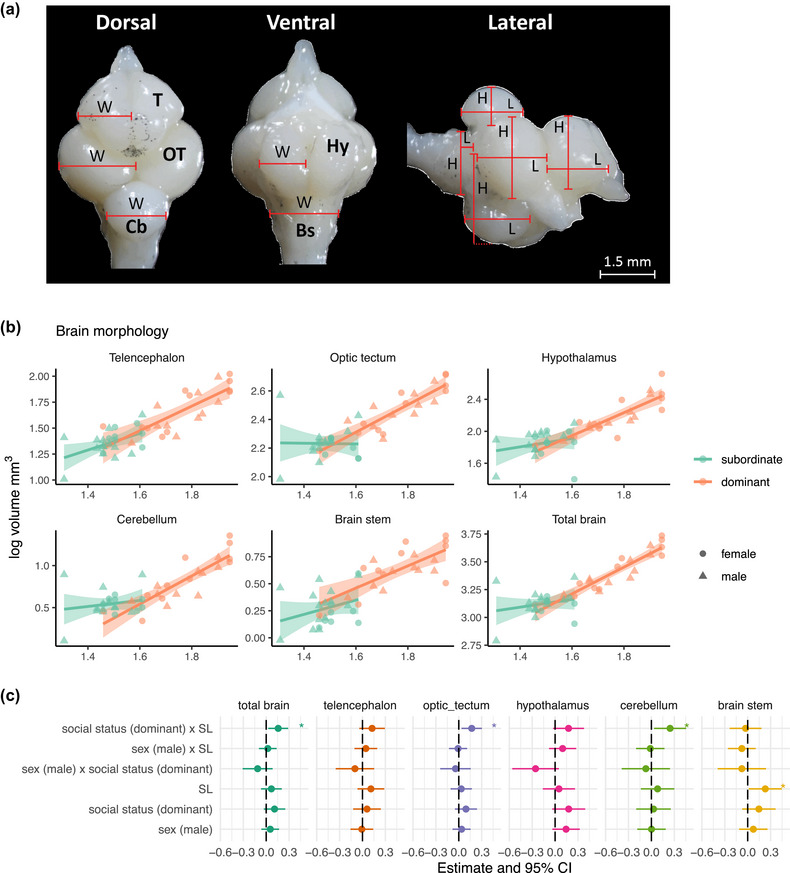
Brain morphology. (a) Brain images prepared for ellipsoid calculations for brain region volumes: T, telencephalon; OT: optic tectum; Cb: cerebellum; Hy, hypothalamus; Bs, brain stem; W, width; H, height; L, length. (b) Regression line and 95% CI of log‐transformed brain measurements on log‐transformed and standardized body size (SL) (*N* = 43). (c) Estimate and 95% CI extracted from the statistical models for each brain measurement as a function of social status and sex, and corrected for body size (SL). *p < .05.

Our statistical analyses showed no significant effect of social status nor sex on fish performance in the cognitive tests (Figures 1 and S1 and detailed statistics are in Table S2). For brain morphology, we found an effect of social status on brain allometry (BLMER: *N* = 43, estimate = 0.157, *p* = .02). With post hoc analyses, the brain–body slope for subordinates (*n* = 22) had a value of (estimate [low, high 95% confidence interval], 0.08 [−0.03, 0.19]) while the dominants (*n* = 21) had a slope of 0.24 [0.16, 0.32] with a partial *R* ^2^ of 0.17. It appeared that brain region sizes were driving these slope differences. Particularly, we found that the regions optic tectum and cerebellum also had significantly steeper slopes in dominants than subordinates (optic tectum: subordinates (0.03 [−0.08, 0.14]), dominants (0.20 [0.11, 0.28]), partial *R* ^2^ = 0.18); cerebellum: subordinates (0.07 [−0.10, 0.25]), dominants (0.32 [0.19, 0.45]), partial *R* ^2^ = 0.16) (Figure 2, detailed statistics are in Table S3). The other brain regions (telencephalon, hypothalamus, and brain stem) did not show significant differences for dominants versus subordinates. Additionally, we did not detect sexual dimorphism in the brain morphology of the tested fish.

**FIGURE 3:**
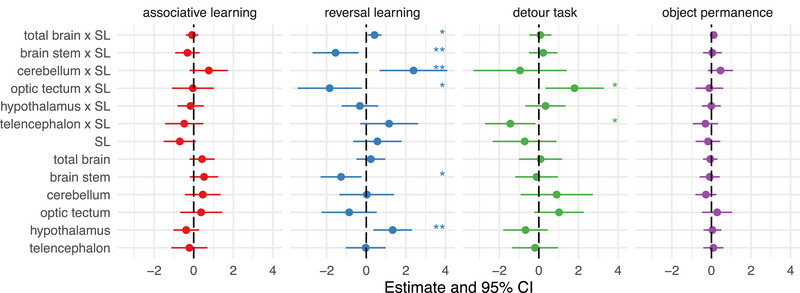
Estimates and 95% confidence from statistical models testing the relationship between brain morphology and cognitive performance. *p < .05; **p < .01.

In the analyses looking into whether brain morphology correlates with individual performance in the cognitive tests, we found significant effects in reversal learning and detour performances, but not for associative learning or object permanence performances. Total brain size relative to body size correlated positively with reversal learning performance (coxme: 0.417 [0.06, 0.77], *p* = .02). Different brain regions appeared to be driving this outcome. On the one hand, reversal learning performance correlated positively with cerebellum size relative to body size (coxme: 0.417 [0.68, 4.10], *p* = .02), and with absolute hypothalamus size (coxme: 0.417 [0.68, 4.10], *p* = .02). On the other hand, the performance in this task correlated negatively with optic tectum and brain stem relative sizes to body size (coxme: optic tectum: −1.850 [−3.47, −0.23], *p *= .025; brain stem: −1.554 [−2.71, −0.39], *p *= .009) (Figure 3, detailed statistics are in Table S4).

In the detour task, the telencephalon and optic tectum had opposite relationships with performance. While the performance correlated positively with optic tectum relative size to body size (BGLMER: 1.803 [0.33, 3.28], *p* = .017), it had a negative correlation with telencephalon relative size (BGLMER: −1.442 [−2.71, −0.17], *p* = .026) (marginal *R* ^2^ = 0.42, conditional *R* ^2^ = 0.67) (Figure 3, detailed statistics are in Table S4).

## 4 DISCUSSION

Across three tests of executive functions, reversal learning, detour, and object permanence tasks, dominants and subordinates of *N. pulcher* performed similarly, with no sex differences in their performance. Yet, social dominance hierarchy had notably an effect on brain allometry, where dominants had steeper brain–body regression slope than subordinates, mostly driven by the optic tectum and cerebellum brain regions. Moreover, brain morphology correlated with individual performance in reversal learning and detour tasks.

To our knowledge, no other studies have explored the performance of dominants and subordinates in the three main executive functions: cognitive flexibility, inhibitory control, and working memory. The existing studies primarily focused on testing for spatial abilities, where the evidence points toward dominants having improved spatial skills than subordinates, like in pheasants, meadow voles and chickadees (Langley et al., 2018; Pravosudov et al., 2003; Spritzer et al., 2004). Yet, in another African cichlid fish, *Astatotilapia burtoni*, with dynamic social hierarchy, dominance does not improve this spatial faculty. However, it seems that social status has a strong influence on social competence in this species. Fish that ascend in social status are more socially competent, which means they are better at using social information to perform optimal behaviors (Wallace et al., 2022). Based on our results, dominance hierarchy did not predict the performance in the three executive function tests. This is in line with the findings of Wallace et al. (2022) and suggests that social hierarchy might only be linked to performance related to social cognition. It is worth noting that the executive function tests we conducted did not involve any social interaction, as animals were only exposed to nonsocial cues. Combining social and nonsocial tests in future research can help to get a better understanding of this issue.

Although social status might not have a significant impact on executive functions, it shows a correlation with brain allometry in *N. pulcher*. The subordinates had an almost flat slope of brain–body size regression, with a value of approximately 0.08, while the dominants had a slope of 0.24. In other words, as subordinates grew, their brains did not grow proportionally, whereas dominants' brains did, to some extent. We speculate two possible alternative explanations for such observations. The first explanation is based on *N. pulcher*’s strategic growth pattern that depends on its status. Subordinates remain smaller than dominants to avoid aggression and eviction from the group (Hamilton et al., 2005; Jungwirth et al., 2023). This suggests they might also be strategic in their energy expenditure on expensive tissues such as the brain and would only start investing in the brain once they become dominants. The second alternative explanation suggests that subordinates are constrained by the presence of dominants. Moreover, it seems that the difference in brain allometry between dominants and subordinates is mainly due to two regions of the brain: the optic tectum, which is typically the largest brain region in teleost, and the cerebellum, which is the most densely packed with cells compared to other brain parts (Van Essen et al., 2018). While there is no concrete evidence, it is possible that either these two areas require more energy to grow or that the other regions, like the telencephalon and hypothalamus, are given a higher priority for energy investment under constraints. Nevertheless, it remains unclear to what extent social dominance affects brain development in cichlids or if their brain allometry follows a nonlinear trajectory. To explicitly differentiate the effects of body size and social dominance hierarchy, experimental testing involving the formation of social groups with fish of various sizes is necessary. This would enable the sampling of dominants and subordinates of similar body sizes and provide a more comprehensive understanding of the relationship between social dominance and brain development in cichlids.

Currently, there is limited research on the brain structure of individuals in dominant and subordinate positions. Nevertheless, there is evidence showing a correlation between social dominance hierarchy and the level of neuronal activity and chemical components in some brain nuclei. For instance, dominant and subordinate fish can have different neural pathway activations, such as neuropeptides and monoamines (Reddon Adam et al., 2015; Winberg et al., 2008), as well as the activation of the social decision‐making network (Maruska et al., 2013). Despite the advances regarding social status’ impact on brain activity, there is still a need for further studies to address the connection between morphology and functionality. Ultimately, this will help us understand how the interplay between morphology and functionality affects cognitive abilities and social behavior.

Our study yielded an interesting finding that brain morphology was correlated with individual performance in two tasks: reversal learning and detour. After statistically correcting for fish body size, which is crucial because dominants were bigger than subordinates, total brain size and cerebellum correlated positively with reversal learning performance. In contrast, optic tectum and brain stem were negatively associated with this task performance. Although evidence suggests that enlarged brains facilitate cognitive flexibility across different species (Buechel et al., 2018; Deaner et al., 2007), little is known about how specific brain region sizes are associated with performance. Based on the limited research available from guppies, large telencephalons often facilitate individual performance in this task (Triki, Granell‐Ruiz et al., 2022; Triki et al., 2024, 2023). Our study suggests that brain regions other than the telencephalon might also play a significant role in cognitive flexibility. Specifically, the cerebellum seems to be important (Butler & Hodos, 2005) in *N. pulcher*. These findings suggest that having more neural tissue in the cerebellum might enhance the potential for acquiring and processing information about updating an existing decision rule.

In the detour task, the fish with larger optic tectum and smaller telencephalons showed better inhibitory control performance. As the optic tectum is responsible for visual sensory perception (Northmore, 2011) and also provides motoric input directly to the hindbrain (Helmbrecht et al., 2018). It can be inferred that those with better visual processing abilities detoured more correctly without touching the cylinder. However, it was unexpected to find a negative correlation between the telencephalon size and performance in this task, given that guppies that have been artificially selected for larger telencephalons show improved performance in detour tests (Triki, Fong et al., 2022, Triki et al., 2023). Different species with different ecologies might have varying relationships between brain morphology and cognitive performance. Additionally, different brain regions and nuclei with distinct functionalities might contribute varying levels of cognitive performance. Animal cognition can be broadly defined as the ability to take information through the senses, process, retain and act on it (Shettleworth, 2001). Hence, each of the main teleost brain regions and their nuclei, including the sensory and motor centers, can play a cognitive role.

There was no difference in fish performance in the associative learning test across social status and sex, and it did not correlate with brain morphology. We did not expect to find a correlation relationship since forming simple associations does not necessarily require complex processing, as even box jellyfish lacking a central nervous system can perform well in such tests (Bielecki et al., 2023). In contrast, an important result was that fish did not perform above chance in the object permanence test. Our study is the third to test fish object permanence abilities, and it seems that fish tend to perform at a chance level with 50% success (Aellen et al., 2022; Triki et al., 2023). Only guppies artificially selected to have larger telencephalons performed relatively better with 60% success (Triki et al., 2023). It is possible that success in object permanence tasks poses cognitive challenges and requires not only substantial working memory but also the ability to create a mental image of an object out of sight (Call, 2001; Lowe et al., 2009).

*N. pulcher* does not appear to have sex‐specific selective pressures that cause differences in gross brain morphology and executive functions between males and females. In another study by La Loggia et al. (2022) that examined *N. pulcher*’s transitive inference abilities, no sex differences were observed either. It is possible that our captive conditions have relaxed sex‐specific selection on the brain and cognitive abilities. To confirm or reject this hypothesis, we need to conduct studies of cognition and brains in both wild and captive‐bred fish (Bshary & Triki, 2022). Furthermore, teleost fish can exhibit finer‐scale sexually dimorphic features, such as differences in the size of brain nuclei, functionality, and various neurohormonal pathways (Godwin, 2010; Okuda et al., 1998). Interestingly, these potential finer‐scale differences between males and females (Aubin‐Horth et al., 2007; Reddon Adam et al., 2015) did not necessarily translate into differences in gross morphology or cognitive performance in our studied species.

In conclusion, our findings highlight that *N. pulcher* performance in executive function tasks may not be linked to group‐level characteristics like social status and sex, but it correlated with individual brain morphology. Thus, what was previously considered as mere noise around the population mean can now be attributed to individual neural traits. Furthermore, depending on species, executive functions in fishes are associated with the size of different brain regions, like telencephalon, optic tectum and cerebellum. Exploring species and individual‐level cognitive performance and linking it to brain morphology is a crucial step toward advancing the field of cognitive sciences.

## AUTHOR CONTRIBUTIONS

**Angelo Guadagno**: Data curation; investigation; validation; visualization; writing—original draft; writing—review and editing; formal analysis. **Zegni Triki**: Conceptualization; methodology; investigation; validation; formal analysis; supervision; funding acquisition; visualization; project administration; resources; writing—original draft; writing—review and editing.

## CONFLICT OF INTEREST STATEMENT

The authors declare that they have no conflict of interest.

### CODE AVAILABILITY STATEMENT

Analyses reported in this article can be reproduced using code archived at the Figshare data repository https://doi.org/10.6084/m9.figshare.24099840.

### PEER REVIEW

The peer review history for this article is available at https://publons.com/publon/10.1002/brb3.3484.

## Supporting information

Supporting information

Video S1. Associative and reversal learning trials with color discrimination test. A fish is presented with two choices (yellow and red), and only choosing the correct choice earns it a food reward given by the experimenter with a plastic pipette.

Video S2. A Cylinder detour task. A transparent cylinder open on either side offers a food reward inside.

Video S3. Object permanence task. Fish watches an object being placed and disappearing behind an opaque screen. The fish is then allowed to enter the test arena, and the experimenter gives a food reward when the fish successfully locates the object.

## Data Availability

Source data from this study are archived in the Figshare data repository https://doi.org/10.6084/m9.figshare.24099840.
