# Supplementary material for: Executive functions and brain morphology of male and female dominant and subordinate cichlid fish
Source: Brain Behav. 2024 Apr 29;14(5):e3484. doi: 10.1002/brb3.3484 (PMC11056711; doi:10.1002/brb3.3484)
Supplement: Supplementary file 1 — Supporting information [file BRB3-14-e3484-s003.docx]

**Electronic supplementary material for: Executive functions and brain morphology of male and female dominant and subordinate cichlid fish**

**Authors:** Angelo Guadagno^*^ and Zegni Triki^*^

**Author affiliation:**

Institute of Ecology and Evolution, University of Bern, Baltzerstrasse 6, 3012 Bern, Switzerland

**^*^Corresponding author:** Angelo Guadagno, [angelo.guadagno@unibe.ch](mailto:angelo.guadagno@unibe.ch); Zegni Triki. [zegni.triki@gmail.com](mailto:zegni.triki@gmail.com)

**Table S1. Sample size.**

| **Dataset** | **N** | **social status** | **sex** | **n** |
| --- | --- | --- | --- | --- |
| **Brain data and also associative learning data** | 43 | subordinate | female | 8 |
|  |  | dominant | female | 11 |
|  |  | subordinate | male | 14 |
|  |  | dominant | male | 10 |
| **Reversal learning dataset** | 42 | subordinate | female | 8 |
|  |  | dominant | female | 11 |
|  |  | subordinate | male | 14 |
|  |  | dominant | male | 9 |
| **Detour task dataset** | 33 | subordinate | female | 6 |
|  |  | dominant | female | 9 |
|  |  | subordinate | male | 10 |
|  |  | dominant | male | 8 |
| **Object permanence dataset** | 31 | subordinate | female | 7 |
|  |  | dominant | female | 8 |
|  |  | subordinate | male | 9 |
|  |  | dominant | male | 7 |

**Table S2. Statistical summary of the cognitive performance by social status and sex.** Bold text indicate statistically significant findings (*p* ≤ 0.05).

| Model | Predictor | estimate | std.error | p.value | conf.low | conf.high |
| --- | --- | --- | --- | --- | --- | --- |
| associative learning | sex (male) | 0.183 | 0.445 | 0.681 | -0.690 | 1.056 |
|  | social status (dominant) | -0.062 | 0.473 | 0.896 | -0.989 | 0.865 |
|  | sex (male) x social status (dominant) | -0.315 | 0.645 | 0.626 | -1.579 | 0.949 |
| reversal learning | sex (male) | 0.268 | 0.541 | 0.621 | -0.792 | 1.328 |
|  | social status (dominant) | -0.227 | 0.611 | 0.710 | -1.424 | 0.969 |
|  | sex (male) x social status (dominant) | 0.240 | 0.792 | 0.761 | -1.312 | 1.793 |
| detour task | sex (male) | 0.882 | 0.700 | 0.208 | -0.490 | 2.254 |
|  | social status (dominant) | 0.670 | 0.866 | 0.439 | -1.027 | 2.367 |
|  | sex (male) x social status (dominant) | -0.597 | 0.794 | 0.452 | -2.153 | 0.959 |
| object permanence | sex (male) | -0.233 | 0.285 | 0.414 | -0.792 | 0.326 |
|  | social status (dominant) | -0.231 | 0.307 | 0.452 | -0.833 | 0.371 |
|  | sex (male) x social status (dominant) | 0.153 | 0.400 | 0.701 | -0.630 | 0.937 |

| Model | Predictor | estimate | std.error | p.value | conf.low | conf.high |
| --- | --- | --- | --- | --- | --- | --- |
| total brain | sex (male) | 0.053 | 0.057 | 0.356 | -0.063 | 0.169 |
|  | social status (dominant) | 0.109 | 0.069 | 0.124 | -0.031 | 0.250 |
|  | SL | 0.068 | 0.067 | 0.317 | -0.068 | 0.204 |
|  | sex (male) x social status (dominant) | -0.112 | 0.097 | 0.254 | -0.309 | 0.084 |
|  | sex (male) x SL | 0.021 | 0.057 | 0.713 | -0.095 | 0.137 |
|  | **social status (dominant) x SL** | **0.157** | **0.064** | **0.020** | **0.027** | **0.288** |
| brain stem | sex (male) | 0.074 | 0.091 | 0.426 | -0.112 | 0.259 |
|  | social status (dominant) | 0.146 | 0.108 | 0.183 | -0.072 | 0.365 |
|  | SL | 0.230 | 0.107 | 0.038 | 0.013 | 0.447 |
|  | sex (male) x social status (dominant) | -0.077 | 0.156 | 0.625 | -0.394 | 0.240 |
|  | sex (male) x SL | -0.077 | 0.089 | 0.394 | -0.258 | 0.104 |
|  | social status (dominant) x SL | -0.028 | 0.103 | 0.786 | -0.236 | 0.180 |
| cerebellum | sex (male) | 0.001 | 0.092 | 0.988 | -0.185 | 0.188 |
|  | social status (dominant) | 0.028 | 0.111 | 0.805 | -0.199 | 0.254 |
|  | SL | 0.081 | 0.108 | 0.459 | -0.138 | 0.300 |
|  | sex (male) x social status (dominant) | -0.073 | 0.156 | 0.643 | -0.389 | 0.243 |
|  | sex (male) x SL | -0.014 | 0.092 | 0.878 | -0.201 | 0.172 |
|  | **social status (dominant) x SL** | **0.245** | **0.103** | **0.024** | **0.035** | **0.455** |
| hypothalamus | sex (male) | 0.144 | 0.088 | 0.114 | -0.036 | 0.323 |
|  | social status (dominant) | 0.178 | 0.106 | 0.103 | -0.038 | 0.394 |
|  | SL | 0.050 | 0.104 | 0.630 | -0.160 | 0.261 |
|  | sex (male) x social status (dominant) | -0.256 | 0.150 | 0.098 | -0.562 | 0.049 |
|  | sex (male) x SL | 0.098 | 0.088 | 0.269 | -0.080 | 0.276 |
|  | social status (dominant) x SL | 0.176 | 0.100 | 0.086 | -0.026 | 0.379 |
| optic tectum | sex (male) | 0.036 | 0.058 | 0.537 | -0.082 | 0.154 |
|  | social status (dominant) | 0.094 | 0.071 | 0.196 | -0.051 | 0.239 |
|  | SL | 0.032 | 0.069 | 0.646 | -0.108 | 0.171 |
|  | sex (male) x social status (dominant) | -0.044 | 0.098 | 0.660 | -0.244 | 0.156 |
|  | sex (male) x SL | -0.010 | 0.059 | 0.870 | -0.129 | 0.109 |
|  | **social status (dominant) x SL** | **0.170** | **0.066** | **0.014** | **0.036** | **0.303** |
| telencephalon | sex (male) | -0.009 | 0.073 | 0.906 | -0.157 | 0.140 |
|  | social status (dominant) | 0.059 | 0.089 | 0.512 | -0.122 | 0.240 |
|  | SL | 0.110 | 0.086 | 0.209 | -0.065 | 0.285 |
|  | sex (male) x social status (dominant) | -0.099 | 0.124 | 0.431 | -0.351 | 0.153 |
|  | sex (male) x SL | 0.044 | 0.073 | 0.554 | -0.105 | 0.193 |
|  | social status (dominant) x SL | 0.126 | 0.083 | 0.137 | -0.042 | 0.294 |

**Table S3. Statistical summary of brain morphology by social status and sex.** Bold text indicate statistically significant findings (*p* ≤ 0.05).

**Table S4. Statistical summary of the individual cognitive performance and brain morphology.** Bold text indicate statistically significant findings (*p* ≤ 0.05).

| Model | Predictor | estimate | std.error | p.value | conf.low | conf.high |
| --- | --- | --- | --- | --- | --- | --- |
| associative learning | telencephalon | -0.218 | 0.465 | 0.640 | -1.129 | 0.693 |
|  | hypothalamus | -0.380 | 0.330 | 0.250 | -1.027 | 0.268 |
|  | optic tectum | 0.374 | 0.539 | 0.488 | -0.683 | 1.431 |
|  | cerebellum | 0.461 | 0.461 | 0.317 | -0.442 | 1.364 |
|  | brain stem | 0.521 | 0.366 | 0.154 | -0.195 | 1.238 |
|  | SL | -0.705 | 0.413 | 0.088 | -1.516 | 0.105 |
|  | telencephalon x SL | -0.478 | 0.492 | 0.331 | -1.442 | 0.486 |
|  | hypothalamus x SL | -0.160 | 0.340 | 0.638 | -0.826 | 0.506 |
|  | optic tectum x SL | -0.043 | 0.540 | 0.937 | -1.100 | 1.015 |
|  | cerebellum x SL | 0.764 | 0.495 | 0.123 | -0.206 | 1.734 |
|  | brain stem x SL | -0.315 | 0.317 | 0.319 | -0.936 | 0.305 |
| reversal learning | telencephalon | -0.028 | 0.516 | 0.957 | -1.039 | 0.983 |
|  | **hypothalamus** | **1.334** | **0.500** | **0.008** | **0.353** | **2.314** |
|  | optic tectum | -0.864 | 0.710 | 0.224 | -2.256 | 0.529 |
|  | cerebellum | 0.026 | 0.704 | 0.970 | -1.354 | 1.406 |
|  | **brain stem** | **-1.275** | **0.526** | **0.015** | **-2.306** | **-0.244** |
|  | SL | 0.562 | 0.625 | 0.368 | -0.662 | 1.787 |
|  | telencephalon x SL | 1.155 | 0.746 | 0.121 | -0.306 | 2.616 |
|  | hypothalamus x SL | -0.324 | 0.471 | 0.492 | -1.246 | 0.599 |
|  | **optic tectum x SL** | **-1.850** | **0.825** | **0.025** | **-3.468** | **-0.233** |
|  | **cerebellum x SL** | **2.388** | **0.872** | **0.006** | **0.678** | **4.097** |
|  | **brain stem x SL** | **-1.554** | **0.592** | **0.009** | **-2.715** | **-0.393** |
| detour task | telencephalon | -0.194 | 0.592 | 0.743 | -1.353 | 0.966 |
|  | hypothalamus | -0.675 | 0.572 | 0.238 | -1.797 | 0.447 |
|  | optic tectum | 1.019 | 0.635 | 0.108 | -0.225 | 2.263 |
|  | cerebellum | 0.900 | 0.932 | 0.334 | -0.927 | 2.727 |
|  | brain stem | -0.115 | 0.549 | 0.834 | -1.191 | 0.961 |
|  | SL | -0.717 | 0.820 | 0.382 | -2.324 | 0.890 |
|  | **telencephalon x SL** | **-1.442** | **0.650** | **0.026** | **-2.715** | **-0.169** |
|  | hypothalamus x SL | 0.338 | 0.517 | 0.513 | -0.676 | 1.353 |
|  | **optic tectum x SL** | **1.803** | **0.752** | **0.017** | **0.329** | **3.277** |
|  | cerebellum x SL | -0.951 | 1.200 | 0.428 | -3.302 | 1.401 |
|  | brain stem x SL | 0.220 | 0.366 | 0.548 | -0.497 | 0.937 |
| object permanence | telencephalon | 0.099 | 0.259 | 0.702 | -0.409 | 0.608 |
|  | hypothalamus | 0.052 | 0.229 | 0.820 | -0.397 | 0.502 |
|  | optic tectum | 0.288 | 0.387 | 0.456 | -0.470 | 1.046 |
|  | cerebellum | -0.279 | 0.271 | 0.303 | -0.809 | 0.252 |
|  | brain stem | -0.088 | 0.260 | 0.734 | -0.598 | 0.421 |
|  | SL | -0.176 | 0.317 | 0.579 | -0.798 | 0.446 |
|  | telencephalon x SL | -0.306 | 0.328 | 0.350 | -0.949 | 0.336 |
|  | hypothalamus x SL | -0.007 | 0.244 | 0.976 | -0.486 | 0.471 |
|  | optic tectum x SL | -0.106 | 0.358 | 0.766 | -0.808 | 0.595 |
|  | cerebellum x SL | 0.459 | 0.322 | 0.154 | -0.172 | 1.091 |
|  | brain stem x SL | 0.045 | 0.243 | 0.853 | -0.432 | 0.522 |
| associative learning | total brain | 0.423 | 0.322 | 0.190 | -0.209 | 1.055 |
|  | total brain x SL | -0.081 | 0.162 | 0.615 | -0.398 | 0.236 |
| reversal learning | total brain | 0.234 | 0.373 | 0.530 | -0.496 | 0.965 |
|  | **total brain x SL** | **0.417** | **0.180** | **0.020** | **0.065** | **0.770** |
| detour task | total brain | 0.088 | 0.555 | 0.874 | -1.000 | 1.176 |
|  | total brain x SL | 0.076 | 0.282 | 0.788 | -0.476 | 0.628 |
| object permanence | total brain | -0.058 | 0.186 | 0.756 | -0.422 | 0.306 |
|  | total brain x SL | 0.114 | 0.093 | 0.219 | -0.068 | 0.296 |

**Figure S1. Learning performance in the (a) associative and (b) reversal tasks.** Dotplot of the fish that successfully reached the learning criterion during the tests. The plot doesn’t show those who failed the test. In the associative test, one male dominant failed the test. In the reversal learning test, 3 subordinate females, 4 dominant females, 3 subordinate males and 2 dominant males failed the task.
